# Functional Trajectories for People With Dementia and Other Comorbidities in the Last Years of Life

**DOI:** 10.1093/geroni/igab046.602

**Published:** 2021-12-17

**Authors:** Ila Broyles, Lauren Palmer, Emily Graf, Qinghua Li, Zhanlian Feng, Helen Lamont, Judith Dey, Iara Oliveira

**Affiliations:** 1 RTI International, RTI International, North Carolina, United States; 2 RTI International, RTI International, Massachusetts, United States; 3 UC Berkeley, Berkeley, California, United States; 4 RTI International, Research Triangle Park, North Carolina, United States; 5 Office of the Assistant Secretary for Planning and Evaluation, Washington, District of Columbia, United States

## Abstract

A hallmark of end-of-life (EOL) is reduced functional ability. However, the impact of dementia and other comorbidities on EOL decline for older adults is less well understood. We estimated the effect of having dementia and comorbidities on activity of daily living (ADL) scores using the 2000-2012 Health and Retirement Study. We identified 5,853 people over age 65 who died and predicted monthly ADL impairments in the last 4 years of life controlling for dementia and other characteristics. Stroke and obesity were associated with significantly higher ADL scores, regardless of dementia status. However, if both dementia and either stroke or obesity were present, dementia was associated with significantly higher ADL scores approximately 1-4 years before death. Functional decline occurred closer to death if they had these conditions and no dementia. Differences in function when patients have dementia and comorbidities may affect understanding of survival time and access to appropriate care.

